# [6]-Gingerol Induces Caspase-Dependent Apoptosis and Prevents PMA-Induced Proliferation in Colon Cancer Cells by Inhibiting MAPK/AP-1 Signaling

**DOI:** 10.1371/journal.pone.0104401

**Published:** 2014-08-26

**Authors:** EK Radhakrishnan, Smitha V. Bava, Sai Shyam Narayanan, Lekshmi R. Nath, Arun Kumar T. Thulasidasan, Eppurathu Vasudevan Soniya, Ruby John Anto

**Affiliations:** 1 Division of Plant Molecular Biology, Rajiv Gandhi Centre for Biotechnology, Thiruvananthapuram, Kerala, India; 2 Division of Cancer Research, Rajiv Gandhi Centre for Biotechnology, Thiruvananthapuram, Kerala, India; Jawaharlal Nehru University, India

## Abstract

We report mechanism-based evidence for the anticancer and chemopreventive efficacy of [6]-gingerol, the major active principle of the medicinal plant, Ginger (*Zingiber officinale*), in colon cancer cells. The compound was evaluated in two human colon cancer cell lines for its cytotoxic effect and the most sensitive cell line, SW-480, was selected for the mechanistic evaluation of its anticancer and chemopreventive efficacy. The non-toxic nature of [6]-gingerol was confirmed by viability assays on rapidly dividing normal mouse colon cells. [6]-gingerol inhibited cell proliferation and induced apoptosis as evidenced by externalization of phosphatidyl serine in SW-480, while the normal colon cells were unaffected. Sensitivity to [6]-gingerol in SW-480 cells was associated with activation of caspases 8, 9, 3 &7 and cleavage of PARP, which attests induction of apoptotic cell death. Mechanistically, [6]-gingerol down-regulated Phorbol Myristate Acetate (PMA) induced phosphorylation of ERK1/2 and JNK MAP kinases and activation of AP-1 transcription factor, but had only little effects on phosphorylation of p38 MAP kinase and activation of NF-kappa B. Additionally, it complemented the inhibitors of either ERK1/2 or JNK MAP kinase in bringing down the PMA-induced cell proliferation in SW-480 cells. We report the inhibition of ERK1/2/JNK/AP-1 pathway as a possible mechanism behind the anticancer as well as chemopreventive efficacy of [6]-gingerol against colon cancer.

## Introduction

Colon cancer is the third most diagnosed type of cancer and the third leading cause of cancer-related mortality in United States. Globally, it is the fourth most common cause of mortality due to cancer. The variations in dietary pattern and increased consumptions of red and processed meat contribute to this increase in incidence of colon cancer [1], [2].Surgical procedures and chemotherapy constitutes the major therapeutic regimens for colon cancer [3]. The increase in drug resistance impedes the treatment of colon cancer and the problems associated with metastasis increases its severity. Search is on for novel therapeutic agents that targets molecular signaling pathways in colon cancer in order to arrest its growth and metastasis.

Ginger (*Zingiber officinale*) has long been used in traditional medicine and is called as “*Maha-aushadhi*” in Ayurveda, meaning “*the great medicine*” [4]. The rhizome from ginger contains many pungent phenolic compounds like [6]-gingerol, [6]-shagol, [6]-paradol and zingerone [5].These compounds have been studied for their anti-bacterial, anti-oxidant, anti-inflammatory and anti-tumor properties [6], [7]. Many studies have proved the anti-cancer properties of these phenolics against cancers of various origins. The rhizome of ginger is an ingredient of daily diets in many countries and is an active ingredient in many traditional systems of herbal medicines, like Oriental medicine and Ayurveda, for managing many ailments including indigestion and other gastrointestinal disorders. This traditional knowledge triggers a particular interest in characterizing the chemo-preventive and anti-cancerous nature of these phenolics against gastric cancers like colorectal cancer or pancreatic cancers.

Among these phenolic compounds, [6]-gingerol (1-[4′-hydroxy-3′-methoxyphenyl]-5-hydroxy-3-decanone) has been studied for its cytotoxic effects in various cancer cell lines, including colorectal cancer. [6]-gingerol was shown to induce cell death in cervical cancer cell line, HeLa, by caspase-3 dependent apoptosis and autophagy [8]. It could inhibit the metastasis of MDA-MB-231 breast cancer cells and induce apoptosis in LNCaP prostate cancer cells [9], [10]. Administration of [6]-gingerol inhibited the growth of several types of murine tumors such as melanomas, renal cell carcinomas and colon carcinomas by enhancing the infiltrations of tumor-infiltrating lymphocytes CD4 and CD8 T-cells and B220^+^ B-cells [11]. [6]-gingerol was also shown to inhibit the progression of phorbol ester-induced skin tumor in ICR mice [12]. Only limited numbers of studies have been published on the anti-cancer properties of [6]-gingerol and its mechanism of action against colon cancer [13], [14], [15].

In this study the cytotoxic effects of [6]-gingerol on SW-480 colon cancer cells were compared with its effects on rapidly dividing normal intestinal epithelial cells from mouse. The study also performed an *in vitro* mechanistic evaluation on the inhibitory effects of [6]-gingerol on phorbol 12-myristate 13-acetate (PMA) induced anti-apoptotic signals in SW-480 cells.

## Materials and Methods

### 2.1. Materials

Dulbecco's modified Eagle's medium (DMEM) was obtained from Life Technologies (Grand Island, NY, USA); Fetal bovine serum (FBS) from PAN Biotech (GmbH, Aidenbach, Germany); Hank's Balanced Salt Solution (HBSS), Epidermal Growth Factor (EGF) and Insulin, Transferrin, Selenium, Sodium Pyruvate solution (ITS-A) from Invitrogen; Antibodies against phospho-p38, phospho-ERK1/2, phospho-JNK, beta-actin and caspases were purchased from Cell Signaling (Beverly, MA, USA) and antibodies against poly ADP ribose polymerase (PARP) was from Santa Cruz Biotechnology (Santa Cruz, CA). [6]-gingerol was purchased from Biomol (Hamburg, Germany). The MAP kinase inhibitors U0126, SP600125, SB203580 and NF-kappaB inhibitor SN50 were procured from Calbiochem (San Diego, CA). All other chemicals, including Phorbol 12-myristate 13-acetate (PMA) were purchased from Sigma Chemicals (St. Louis, MO, USA).

### 2.2. Cell culture

Human colon cancer cell lines, SW-480 and HCT116 were obtained from National Centre for Cell Sciences (NCCS), Pune, India. Cells were cultured in Dulbecco's Modified Eagle's Medium (DMEM) supplemented with 10% Fetal Bovine Serum (FBS) along with 100 U/ml penicillin, 50 microgram/ml streptomycin and 1 microgram/ml of amphotericin B. The cell lines were maintained at 37°C in a humidified atmosphere of 5% CO2 and were sub-cultured twice weekly.

Normal intestinal epithelial cells (IECs) were isolated from mouse colon as per established protocol [16], [17], with appropriate modifications, as approved by the Institutional Animal Ethical Committee, Rajiv Gandhi Centre for Biotechnology as per rules of the *Committee for the Purpose of Control and Supervision of Experiments on Animals*, Ministry of Environment and Forest, Government of India (Sanction No: IAEC/151/RUBY/2012). Briefly, mouse (*Swiss albino*, 7 weeks old, IAEC sanction No: IAEC/151/RUBY/2012) colon was dissected out and cleaned aseptically with 1× Hank's Balanced Salt Solution (HBSS) to remove fecal matter. The intestine was longitudinally opened and cut into 1 cm pieces and were incubated in presence of type 1 collagenase (200 U/ml) for 30 min with vigorous shaking. The dislodged epithelial cells were isolated by centrifuging the supernatant. These cells were cultured in high glucose DMEM with 10% FBS and 2× antibiotics, containing 10 ng/ml epidermal growth factor (EGF) and 5 µg/ml Insulin-Transferrin-Selenium-Sodium Pyruvate (ITS-A) (Invitrogen, USA). This cell line was maintained at 37°C in a humidified atmosphere of 5% CO_2_ and was sub-cultured once in a month.

### 2.3. Drug treatment

[6]-gingerol stock (20 mg/ml) was prepared in ethanol and the working concentrations were prepared by diluting this stock in dimethyl sufoxide (DMSO). For MTT assay, 5×10^3^ cells/well of human colon cancer cells and 10^4^ cells/well of mouse IECs were seeded in 96-well plates. Cells were treated with [6]-gingerol for 48 h,72 h or 96 h before performing MTT assay and for 16 h before Annexin-V staining. PMA (5 mg/ml) was prepared in DMSO and stored at −20°C. In all combination treatments [6]-gingerol was added 2 h before treating with PMA. In cell viability assay/Western blot for combination treatments with MAP kinase inhibitors (2.5 micromolar U0126, 5 micromolar SP600125, 1′micromolar SB203580) or NF-kappaB inhibitor (18 micromolar SN50), cells were first treated with the inhibitor for 1 h and subsequently pre-treated with [6]-gingerol for 2 h, followed by exposure to PMA for 48 h before performing MTT assay. For Electrophoretic Mobility Shift Assay (EMSA), 10^6^ cells of SW-480 were seeded in 60 mm plates and cells were pretreated with [6]-gingerol for 2 h and then with PMA for 30 min.

### 2.4. Cell viability assay

Cytotoxic effects of [6]-gingerol was determined by MTT assay as described earlier [18] and the relative cell viability percentage is expressed as [A_570_ of treated wells/A_570_ of untreated wells ×100].

### 2.5. Annexin V-Propidium Iodine staining

The membrane flip-flop induced by 16 h treatment with [6]-gingerol was analyzed by staining the cells with fluorescein isothiocyanate-conjugated Annexin V/PI (Santa Cruz Biotechnology) according to the manufacturer's instructions and the apoptotic cells were photographed under fluorescent microscope [19]. The total number of cells in the microscopic field was counted under a phase contrast microscope and the number of fluorescent cells in the same field was counted under a fluorescent microscope. The fraction of fluorescent cells to total number of cells is represented as percentage of apoptotic cells. This was repeated in several fields of the same well and the average was taken for plotting.

### 2.6. Immunoblotting

The total protein isolated from cells following treatments, with or without PMA/[6]-gingerol, were subjected to Western blotting as described previously [20]. In short, 60 microgram of whole cell lysate was separated on a 10–15% polyacrylamide gel, blotted on to a PVDF membrane, probed with corresponding antibody and detected using ECL (Millipore, Billerica, MA, USA).

### 2.7. Electrophoretic Mobility Shift Assay (EMSA)

EMSA was performed to evaluate DNA-binding activity of NF-kappa B or AP-1 transcription factors in cells treated with PMA and/or [6]-gingerol, as described earlier [21]. In brief, 10 microgram of nuclear proteins, isolated from cells following drug treatments, were incubated for 30 min with ^32^P-end-labeled double-stranded 45-mer oligonucleotide for NF kappaB (5′TTGTTACAAGGGACTTTCCGCTGGGGACTTTCCAGGGAGGCGTGG-3′; at 37°C) or 21-mer oligonucleotide for AP-1 (5′-CGCTTGATGACTCAGCCGGAA-3′;at 30°C) and the DNA protein complex was resolved on 6.6% non-denaturing polyacrylamide gel, which was dried and visualized on Phosphor Imager (Personal Molecular Imager FX; Bio-Rad Laboratories, Hercules, CA, USA).

### 2.8. Statistical Analysis

All the experiments were performed at least in triplicates (n = 3). The error bars represent ± standard deviation (S.D) of the experiments. The statistical analysis was carried out using Student's t-test and the symbols *, ** and *** represents P-values, p≤0.05, p≤0.005, and p≤0.001 respectively.

## Results

### 3.1. [6]-gingerol induces dose dependent cytotoxicity in colon cancer cells while normal intestinal epithelial cells are unaffected

**Figure 1:**
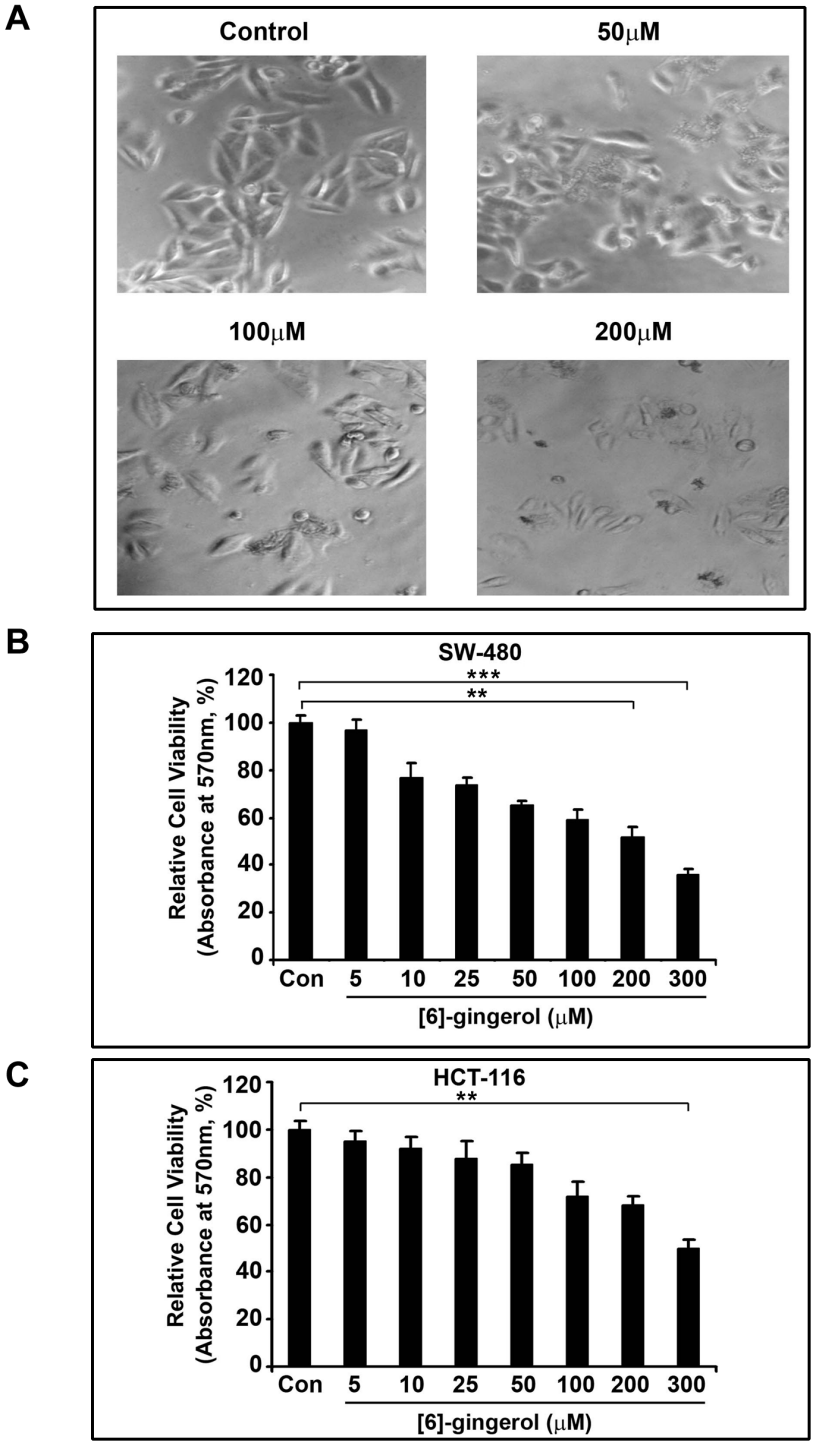
Cytotoxic effects of [6]-gingerol on SW-480 colon cancer cells. (A) Phase contrast microscopy images of morphological changes in SW-480 cells when treated with indicated concentrations of [6]-gingerol for 72 h. (B) Relative cell viability of SW-480 cells after [6]-gingerol treatment, determined by MTT assay and expressed as percentage of the untreated control. 5000 cells/well of SW-480 cells were treated with the indicated concentration of [6]-gingerol for 72 h prior to MTT assay.(C) Relative cell viability of HCT-116 cells after [6]-gingerol treatment. The results are represented as mean of triplicate experiments ± standard deviation (SD).

**Figure 2.Comparison:**
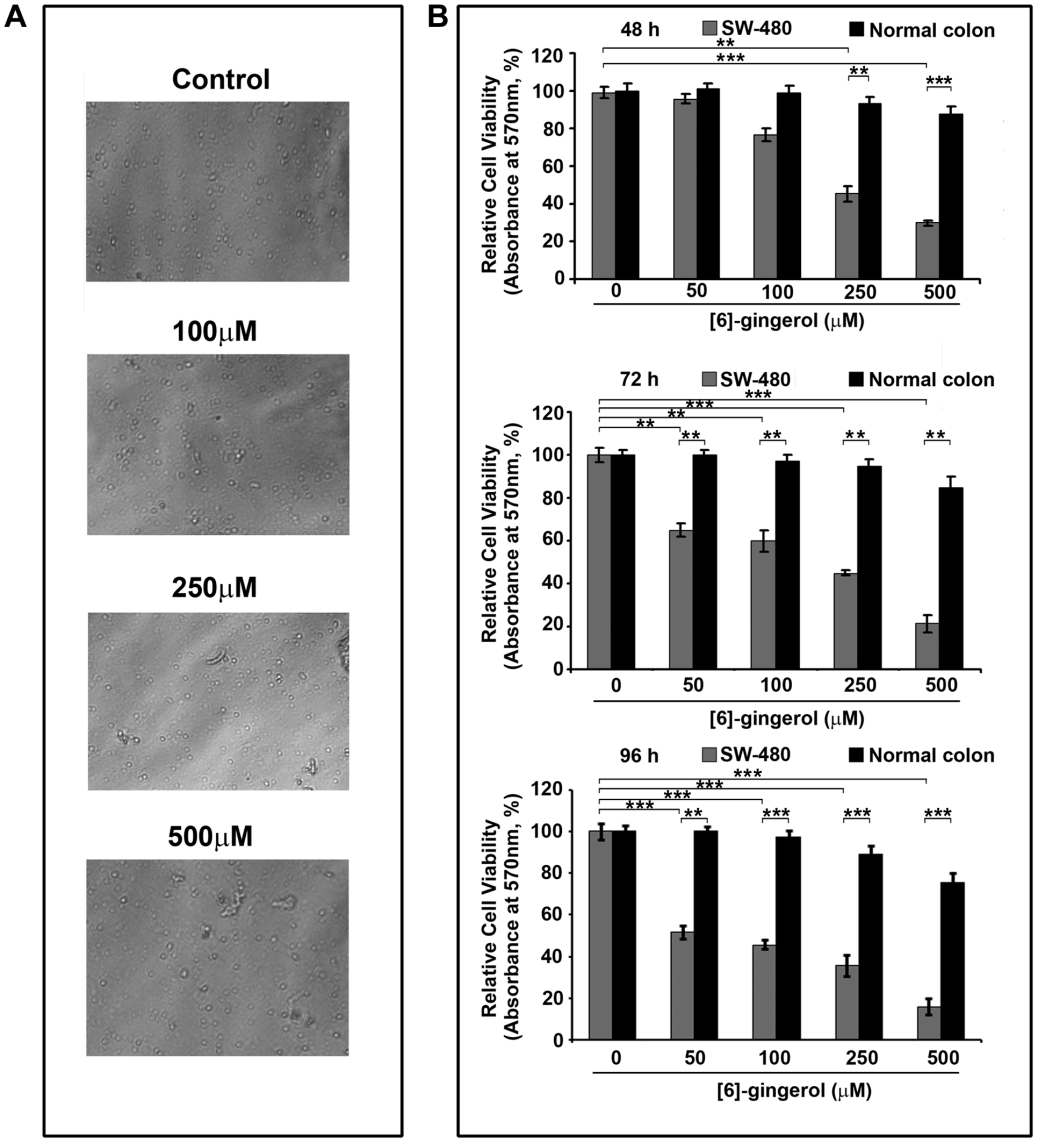
Comparison of cytotoxic effects of [6]-gingerol on SW-480 colon cancer cells with mouse normal intestinal epithelial cells (IECs). (A) Phase contrast microscopy images of IECs treated with indicated concentrations of [6]-gingerol for 72 h. (B) Time dependent cytotoxic effects of [6]-gingerol on SW-480 and IECs at 48, 72 and 96 h. 5000 cells/well of SW-480 and 10,000 cells/well of IECs were treated for 48 to 96 h with indicated doses of [6]-gingerol prior to MTT assay. The results are represented as mean ± SD from triplicate experiments.

[6]-gingerol was screened for its cytotoxic effects on SW-480 and HCT116 cells at various concentrations ranging from 5 micromolar to 300 micromolar, for 72 h, using MTT assay. Dose-dependent changes in cellular morphology were clearly evident under phase contrast microscope (Fig. 1A). The results shows [6]-gingerol to be cytotoxic towards both SW-480 and HCT116 cells in a dose-dependent manner, with prominent cytotoxicity at higher concentrations producing an IC_50_ value of 205±5 micromolar and 283±7 micromolar, respectively (Fig. 1B and Fig. 1C).The effect was more prominent in case of SW-480 than HCT116 and thus the former was used in all further studies. On the contrary, the results from MTT assay with mouse normal IECs revealed only 10–15% cytotoxicity even at twice the IC_50_ concentration of [6]-gingerol against SW-480.The cellular morphology and cell number was seen largely unaffected even at 500 micromolar of [6]-gingerol (Fig. 2A). Studying the time-dependent effect of [6]-gingerol at the effective concentrations revealed an increase in cytotoxicity of SW-480 cells over time from 48 h to 96 h, although the cell viability of mouse normal IECs remained unchanged (Fig. 2B).These results suggest the specificity of [6]-gingerol in inducing cytotoxicity in cancerous cells without being toxic to normal cells even at higher concentrations.

### 3.2. [6]-gingerol induces apoptosis in colon cancer cells, but not in normal intestinal epithelial cells

**Figure 3:**
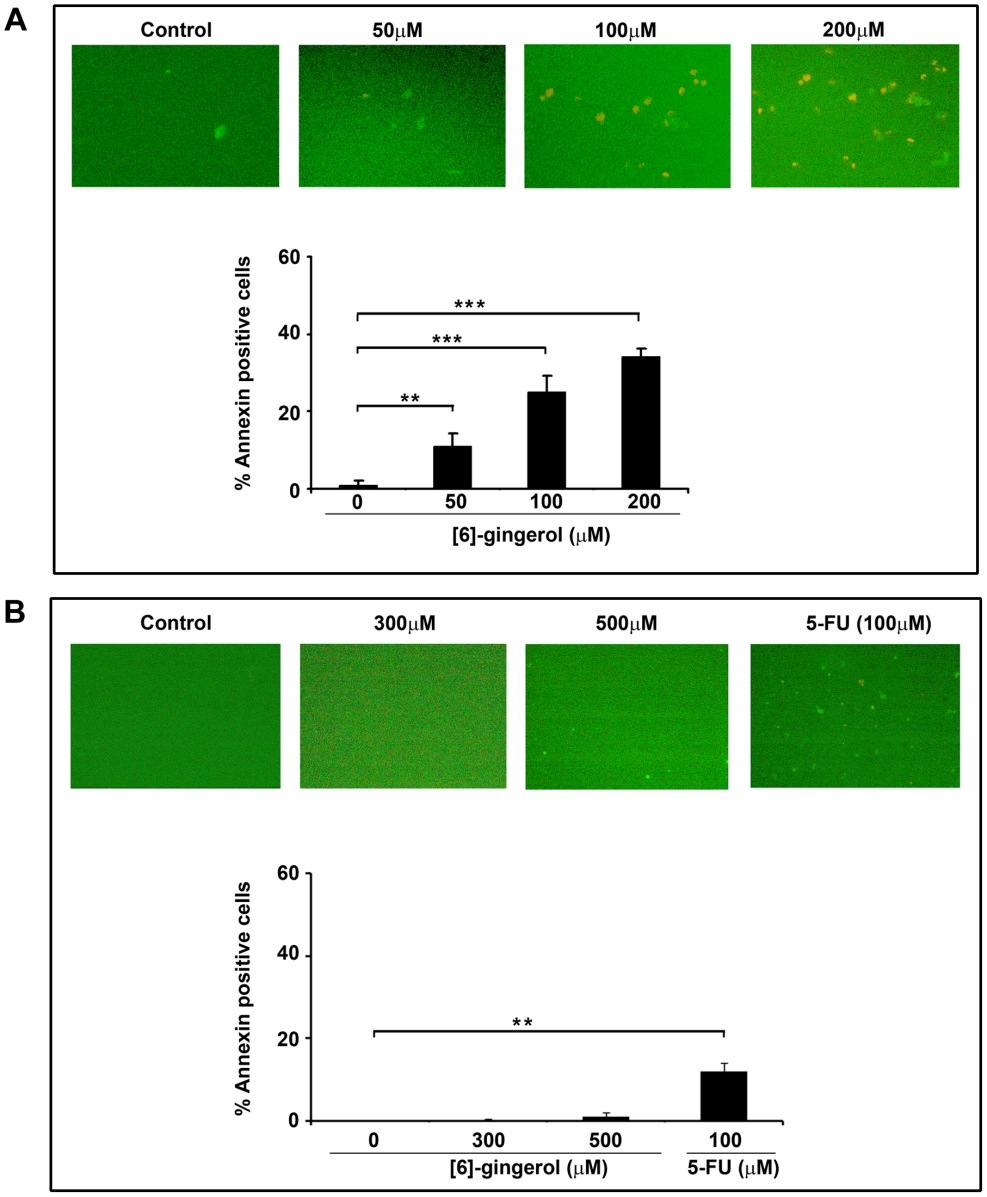
Effects of [6]-gingerol on induction of apoptosis in SW-480 cells and IECs. (A, upper panel) SW-480 cells were treated with indicated concentrations of [6]-gingerol for 16 h and stained for Annexin V/propidium iodide (PI) positivity. Green-fluorescence indicates Annexin V binding to the damaged cell membrane during early apoptosis and the red fluorescence indicates PI binding to the exposed DNA during late apoptosis. (A, lower panel) Graphical representation of the percentage of Annexin V/PI positive SW-480 cells in relation to [6]-gingerol concentration. (B, upper panel) IECs were treated with up to 500 µM of [6]-gingerol for 16 h before performing Annexin V-PI staining. Treatment with 100 µM 5-FU was used as positive control for apoptosis induction. (B, lower panel) Representative histogram of the percentage of Annexin/PI positive IECs following [6]-gingerol/5-FU treatment. The results are represented as mean from triplicate experiments ± SD.

In order to assess whether the induction of cytotoxicity by [6]-gingerol is mediated by apoptosis, the membrane flip-flop and externalization of phosphotedylserine were monitored in [6]-gingerol treated SW-480 cells by performing Annexin-V/PI staining. The results from Annexin-V/PI staining confirmed the early events of apoptotis in [6]-gingerol treated SW-480 cells, as evident from the dose-dependent increase in the fluorescent cells (Fig. 3A).The results from similar study performed on mouse IECs showed only negligible number of fluorescent cells, even at 500 micromolar of [6]-gingerol treatment.100 micromolar 5-Fluro Uracil (5-FU) served as positive control for Annexin V staining on normal IECs (Fig. 3B).

**Figure 4:**
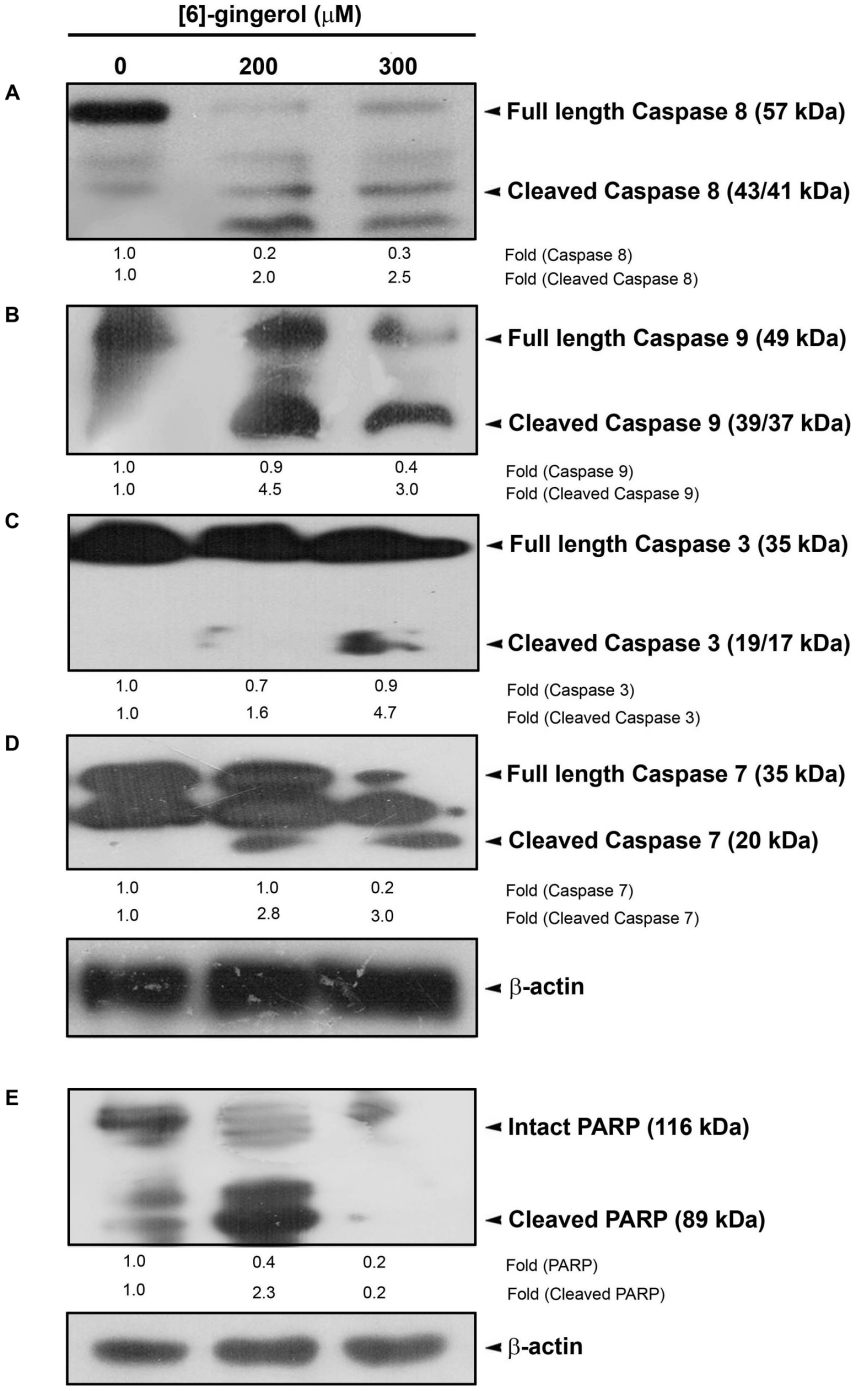
Effects of [6]-gingerol on activation of caspases and PARP cleavage in SW-480 cells. SW-480 cells (10^6^ cells/well) were treated with the indicated concentrations of [6]-gingerol for 48 h and the whole cell extracts were Western blotted on to PVDF membrane. The activation of caspases and PARP cleavage were detected by probing the blotted membrane with antibodies against Caspase-3, 7, 8, 9 and against PARP. The blots were developed using Enhanced Chemiluminescence (ECL). The relative fold differences of bands with control treatments were quantified from volume analysis of the bands using Biorad- Quantity One software. β-actin served as the loading control in each case.

We also analyzed the cell extracts from SW-480 cells treated with [6]-gingerol for the activation of caspases-8, 9, 3, 7 and cleavage of PARP using immunoblotting experiments. Caspases are a family of cysteine proteases which are activated during the apoptotic program. We observed a significant cleavage of pro-caspase 8 to its active fragments (p43/41) and procaspase-9 to its active fragments (p35/37) (Fig. 4A and 4B). The activation of the effector caspases 3 and 7 were also induced by [6]-gingerol in a dose-dependent manner, cleaving procaspase-3 to its active fragments (p17/19) and enhancing the cleavage of procaspase-7 to its active fragment (p20) (Fig. 4C and 4D). Finally, we examined cleavage of the DNA repairing protein, PARP, which is a substrate of caspase-3.Upon treating the cells with 200 and 300 micromolar of [6]-gingerol, the 116-kDa form of PARP was cleaved to the 89 kDa fragment (Fig. 4E), confirming a caspase mediated apoptosis. Since 200 micromolar of [6]-gingerol was efficiently activating caspases leading to PARP cleavage and apoptosis, this concentration was used in further signaling studies.

### 3.3. [6]-gingerol inhibits PMA-induced activation of the MAP kinases in SW-480 cells

**Figure 5:**
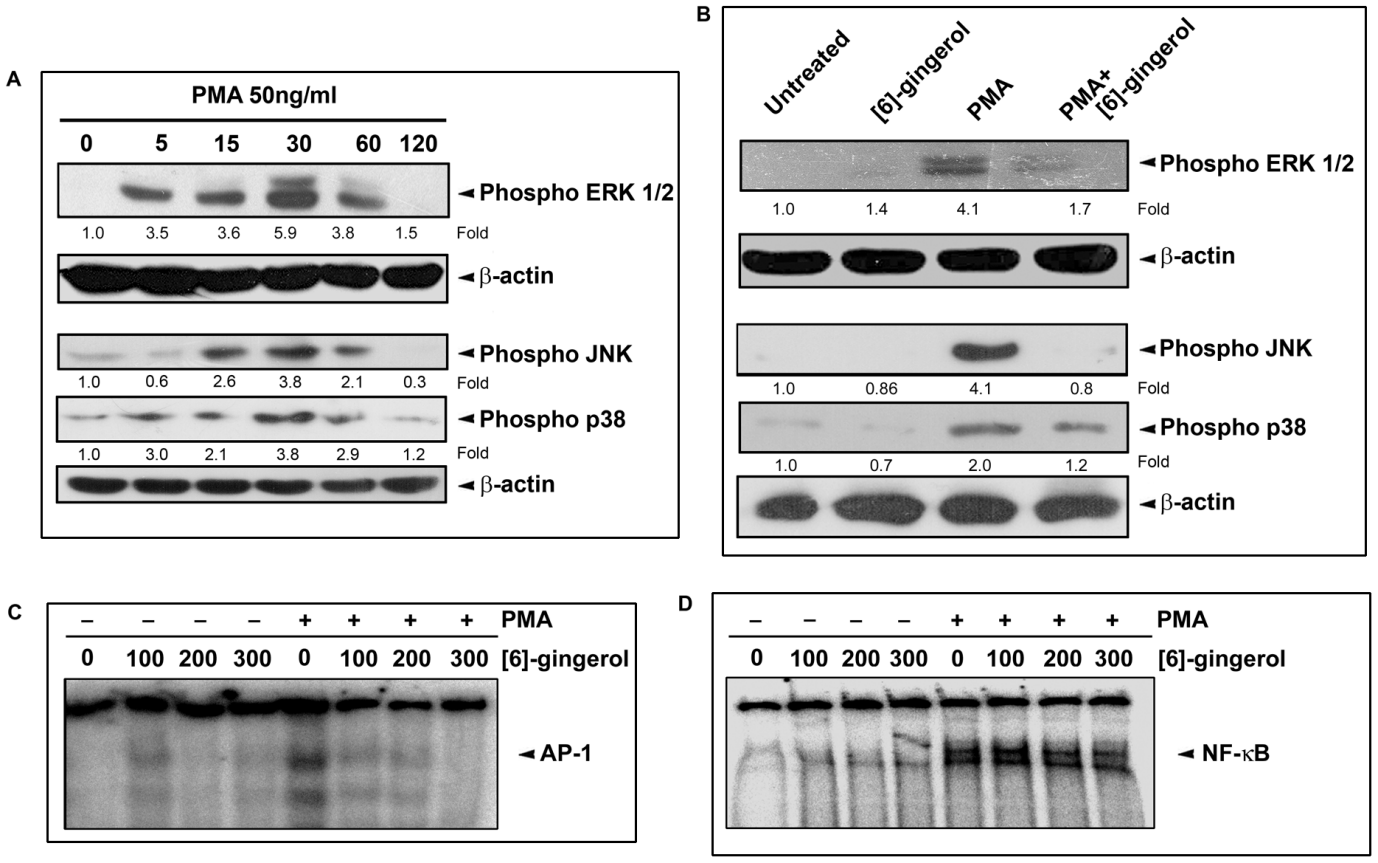
Effect of [6]-gingerol on PMA-induced phosphorylation of MAP kinases and activation of AP-1 and NF-kappaB in SW-480 cells. (A) Overnight grown SW-480 cells were treated with 50 ng/ml of PMA for different time intervals (0–120 min) and the whole cell lysate was immunoblotted onto PVDF membrane, probed using antibodies against phospho-ERK1/2, phospho-JNK and phospho-p38, developed by ECL. The kinetics of PMA-induced phosphorylation of these MAP kinases were followed from this blot (B) SW-480 cells were pre-treated with 200 µM [6]-gingerol before treating with 50 ng/ml of PMA for 30 min and the whole cell lysates were immunoblotted, probed and detected as above. The relative fold difference of bands with control treatments are indicated below each lane. β-actin served as the loading control in each case. (C) SW-480 cells, pre-treated with indicated concentrations of [6]-gingerol for 2 h, were treated with PMA (50 ng/ml) for 30 min. The nuclear extracts from each treatment were analysed for the activation of AP-1 or (D) NF-kappaB, by performing the transcriptional binding assay on isotope labelled AP-1/NF-kappaB specific DNA binding probe and analysing it on electrophoretic mobility shift assay (EMSA). The arrowhead in each case indicates complexes between AP-1/NF-kappaB and DNA probe.

PMA is a well- known tumor promoter that activates almost all protein kinase C (PKC) isozymes, which are prominent upstream regulators of mitogen-activated protein (MAP) kinase pathway [22], [23].The kinetics of phosphorylation of Ras/Raf/extracellular signal-regulated kinase (ERK1/2), c-jun NH 2 -terminal kinase (JNK) and p38 MAP kinase in SW-480 cells in response to treatment with 50 ng/ml (80 nM) PMA for different time intervals were studied. Western blot analysis revealed a time dependant phosphorylation of ERK1/2, JNK and p38.In all three cases, phosphorylation was evident at 5 min from PMA treatment and it peaked at 30 min and then receded by 120 min (Fig. 5A). The effect of [6]-gingerol on the PMA-induced transient phosphorylation of MAP kinases was studied on 30 min PMA-treated SW-480 cells, pretreated with 200 micromolar [6]-gingerol for 2 h. Interestingly, [6] - gingerol pretreatment abolished the PMA-induced transient phosphorylation of ERK1/2 and JNK almost completely, but only partially abolished the phosphorylation of p38 MAP kinase in SW-480 cells (Fig. 5B).

### 3.4. [6]-gingerol inhibits the transcriptional binding activity of PMA-induced AP-1, but not NF-kappaB, in SW-480

PMA is a well known activator of the transcription factors AP-1 and NF-kappaB in different cancer cells [24], [25], [26], [27]. To determine the transcriptional binding activity of AP-1 in SW-480, induced in response to PMA, nuclear extract from the cells treated with 50 ng/microlitre PMA for 1 h was used for EMSA. A clear dose dependent activation of AP-1 was observed in SW-480 cells upon PMA treatment (Fig. 5C; Lane 5).A dose-dependent down-regulation of this PMA-induced AP-1 binding was observed upon 2 h pre-treatment with [6]-gingerol (Fig. 5C; Lane 6–8). [6]-gingerol pretreatment did not show a drastic effect on the transcriptional binding property of NF-kappaB, even though there was a significant inhibition of its DNA binding at 300 micromolar [6]-gingerol (Fig. 5D; Lane 6–8).

### 3.5. [6]-gingerol inhibits the PMA-induced cell proliferation in SW-480 through inhibition of ERK1/2 and JNK MAP kinase pathways

**Figure 6:**
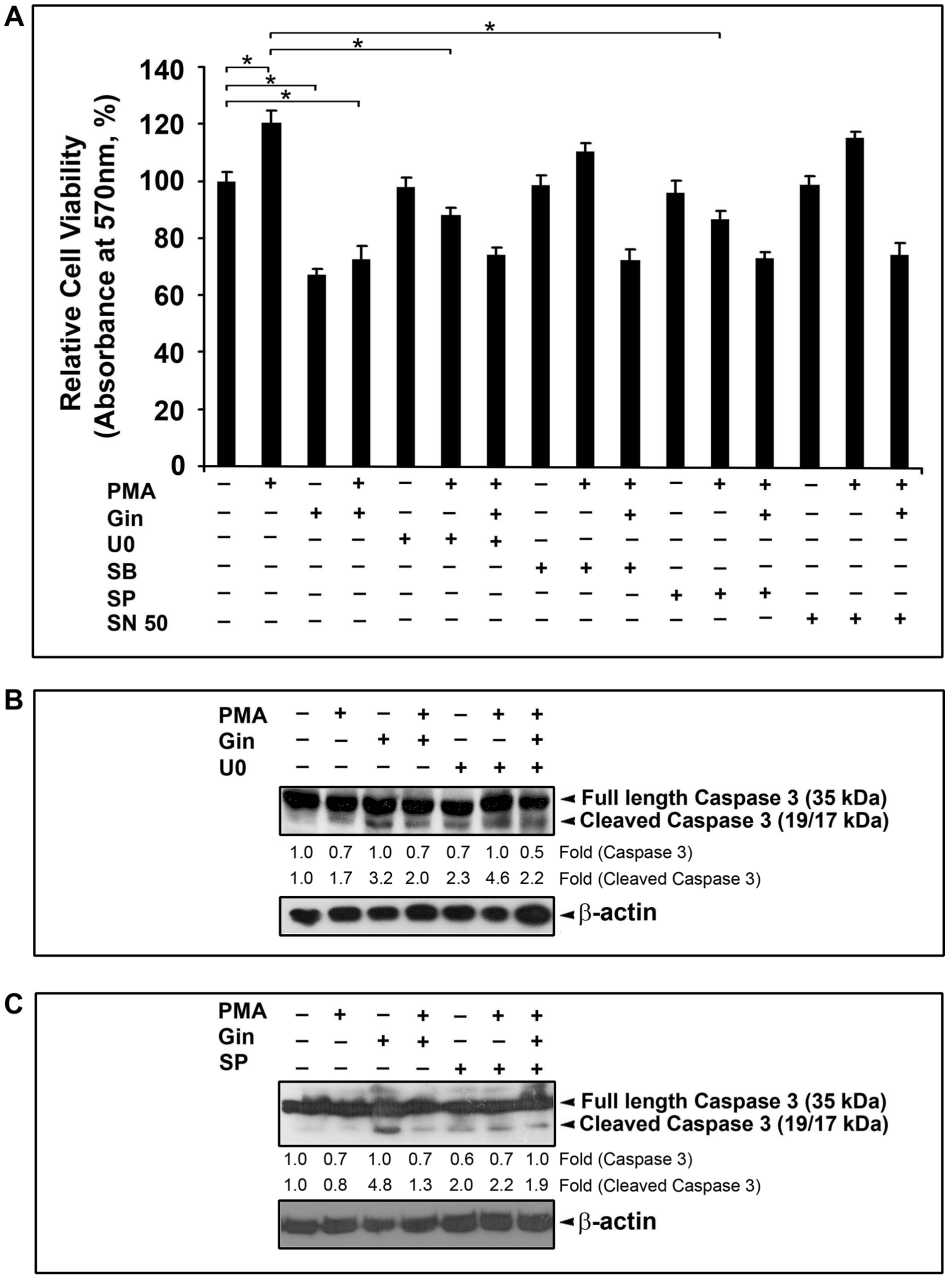
Effects of [6]-gingerol on PMA-induced cell proliferation in SW-480 in presence of inhibitors of MAP kinases and NF-kappaB. (A) Overnight grown SW-480 cells were pre-treated with inhibitors (U0126, SP600125, SB203580 for ERK1/2, JNK and p38 MAP kinases, respectively or SN50 for NF-kappaB) for 1 h and then treated with [6]-gingerol for 2 h, followed by exposure to PMA for 48 h, before performing cell viability assay using MTT. The relative viability of SW-480 cells in each treatment represented as percentage of untreated control. The values presented are mean of triplicate experiments ± SD. Total lysates from cells treated with combination of [6]-gingerol with (B) U0126 and (C) SP600125 inhibitors and from appropriate control treatments were Western blotted and probed with anti-caspase-3 antibody and the blots were developed using ECL. Beta-actin served as the loading control.

In order to further understand the mechanistic details behind the inhibitory effects of [6]-gingerol on PMA-activated signal pathways in SW-480, the viability and proliferation of SW-480 cells were monitored by MTT assay after treating them with various combinations of PMA, [6]-gingerol and inhibitors of MAP kinases or NF-kappaB. Primarily, PMA treatment enhanced the proliferation of SW-480 cells significantly. But, [6]-gingerol pre-treatment of the cells brought down the PMA-induce proliferation drastically. However, viability of SW-480 cells after [6]-gingerol treatment were comparatively higher in presence of PMA (Fig. 6A).

Next, we performed the same experiments on SW-480 cells pre-treated with inhibitors of members of MAP kinase family and NF-kappaB. U0126 was used as a specific inhibitor of ERK1/2 pathway, SP600125 as an inhibitor of JNK activation and SB203580 as the inhibitor of p38 MAP kinase pathway.SN50 was used as an inhibitor of NF-kappaB transcription factor. None of the above inhibitors had any cytotoxic effect on SW-480 cells at the tested concentration (Fig. 6A). But, pre-treatment with U0126 and SP600125 significantly reduced the PMA-induced proliferation of SW-480 cells to the same extend, even though to a lesser extend compared to [6]-gingerol pre-treatment (Fig. 6A). These results prove the essential role of ERK1/2 and JNK MAP kinases pathways in inducing proliferation of SW-480 cells in presence of PMA. Pre-treatment with SB203580 and SN50 had little effect on PMA-induced proliferation of SW-480 cells (Fig. 6A), proving the lack of involvement of p38 MAP kinase pathway and NF-kappaB transcription factor in this process.

Finally, [6]-gingerol treatment was performed on SW-480 cells pre-treated with each of the above inhibitors prior to induction with PMA. Surprisingly, in each case the cell viability was reduced to a level similar to that of pre-treatment with only [6]-gingerol prior to PMA treatment. This result suggests that, in presence of U0126, [6]-gingerol complements this ERK1/2 inhibitor by abrogating the activation of JNK MAP kinase and, in presence of SP600125 it inhibits the ERK1/2 activation, thus reducing cell viability to the same level in both these cases. In presence of SB203580 and SN50 inhibitors [6]-gingerol exerts its inhibitory effects on both ERK1/2 and JNK MAP kinase pathways, thus brings down the cell viability to the same level as above. These results attest the equal contribution of both ERK1/2 and JNK MAP kinase pathways in [6]-gingerol mediated inhibition of PMA-induced cell proliferation of SW-480. Since there is no additive or synergistic inhibitory effects on proliferation of PMA-treated SW-480 cells resulting from pre-treatment with the combination of [6]-gingerol with U0126 or SP600125, compared to pre-treatment with [6]-gingerol alone, ERK1/2 and JNK MAP kinase pathway seems to be the key signaling pathways affected by [6]-gingerol during inhibition of PMA-induced cell proliferation in SW-480.

In order to test whether the inhibitory effects on the PMA-induced cell proliferation of SW-480 cells by [6]-gingerol and U0126 or SP600125 were apoptosis mediated, we monitored the activation of caspase 3 from each of these treatments by Western blotting. We observed cleavage of procaspase 3 in all the treatments involving [6]-gingerol, U0126 or SP600125 and in combination of [6]-gingerol and these inhibitors (Fig. 6B and 6C). It was also interesting to note that no additional degradation of the caspase 3 mother band was produced when the inhibitors and the gingerol were used together. This attests that down-regulation of ERK1/2/JNK/AP-1 pathway induced by PMA, is responsible for the inhibitory effects of [6]-gingerol on PMA-induced cell proliferation in SW-480.

## Discussion

Anti-cancer and anti-inflammatory properties of [6]-gingerol has been recognized since long and many studies have reported different molecular mechanisms behind these properties. The inhibitory effects of [6]-gingerol against cancers of various origins were reported previously. [6]-gingerol has been shown to be particularly effective against skin carcinoma and in inhibiting of angiogenesis in endothelial cells [28], [29]. Ginger being a dietary supplement and a major ingredient in many traditional medicines, it is logical to study the effects of [6]-gingerol on cancer of gastro-intestinal tract such as colon cancer. Moreover, the pharmacokinetic studies on the active components of ginger from orally administered ginger extracts in human beings detected a significant levels of [6]-gingerol, either in free or conjugated form, in plasma and colon tissues [30], [31]. Another study on bioavailability of [6]-gingerol in rats suggested that when orally administered it is detected at concentrations of 4.3 µg/ml in plasma at ten minutes post-administration. The same study also determined its high distribution in tissues with highest concentration in tissues of gastrointestinal tract. The tissue to plasma ratio of [6]-gingerol was reported to be >1 and was attributed to its lipophilicity [32]. All these facts support a study on anti-cancer effects of [6]-gingerol on colon cancer.

The first decade of 21^st^ century had an increased number of studies on characterizing the mechanisms behind the anti-cancer effects of phytochemicals. Studies on the mechanistic evaluation of anti-cancer properties of [6]-gingerol against different kinds of cancers provided valuable insights into its multiple mechanisms of action in bringing about cytotoxic or pro-apoptotic effects in cancer cells. Some recent reports on the anti-cancer activities of [6]-gingerol against colon cancer presented different mechanisms for its action on different colon cancer cell lines [13], [14], [15], [33]. The present study on SW-480 cell line demonstrates the *in vitro* cytotoxicity of [6]-gingerol with an IC_50_ value of 205 micromolar. The previous study on *in vitro* cytotoxic effects of [6]-gingerol on SW-480 cell line reported only 17% cell death at this concentration [13].These differences in the magnitude of effects might be due to the variations in the method used in studying cytotoxicity. It is also noteworthy that the same study reported only 13% cytotoxicity in LoVo cells when treated with 200 micromolar of [6]-gingerol for 72 h, which was later reported in a different study as 75% at 50 micromolar in the same cell line after 48 h treatment [15]. The dose-dependent increase in apoptotic cells (Annexin-V/PI positive cells) in SW-480 cells upon treatment with [6]-gingerol, upto 25-folds at 300 µM concentration, proved that the cytotoxicity was induced mainly by apoptosis. Previous studies reported both cell cycle arrest and apoptosis as the mechanism of action of [6]-gingerol [13], [34]. Two-fold increase in apoptosis was reported at similar conditions in SW-480 by [13], but they also demonstrated significant G2/M arrest in cell cycle in response to [6]-gingerol treatment. Many previous reports suggested that [6]-gingerol induces apoptosis only at or near 300 micromolar in cancer cells [13], [34], [35], [36] and below this concentration it induces cytotoxicity mainly by other mechanisms. However, we observed fluorescent cells in SW-480 treated with even 100 micromolar [6]-gingerol, clearly suggesting early apoptosis events even at lower concentrations. Furthermore, the dose-dependent activation of caspases-8,9, 3 and 7 in our study further confirmed apoptosis as the major mechanism of cell death in SW-480 cells treated with [6]-gingerol. Activation of caspase-9 by [6]-gingerol confirms the involvement of mitochondrial pathway in [6]-gingerol-mediated apoptotis. However, the cleavage of caspase-8 induced by [6]-gingerol may not essentially suggest the involvement of receptor-mediated pathway, as mitochondrial pathway could also lead to cleavage of caspase-8 through cleavage of BH3 interacting-domain death agonist (BID) [37]. Induction of apoptosis in SW-480, a p53-mutant colon cancer cell line, by [6]-gingerol is particularly interesting as p53-mutant cells are considered to be more resistant to standard chemotherapeutics and radiation [13], [36]. p53-independent induction of apoptosis by [6]-gingerol was reported previously in pancreatic cancer cell lines, where the expression of Cyclin-dependent kinase inhibitor, p21^cip1^, was increased independent of p53 expression leading to decrease in Cyclin A and Cyclin-dependent kinase expression and cell cycle arrest [36].

Even though [6]-gingerol is generally considered as non-toxic to normal cells, some of the recent studies reported otherwise. Genotoxic effects of [6]-gingerol at higher doses was demonstrated in human hepatoma G2 cells [38]. Another recent study reported that [6]-gingerol treatment leads to a significant dose-dependent inhibition of proliferation of the dermal papilla cells of human hair follicles and elongation of hair shaft [39].They were successful in demonstrating apoptosis in dermal papilla cells at less than 50 micromolar [6]-gingerol. In light of these studies we studied the cytotoxic effects of [6]-gingerol on cultured mouse intestinal epithelial cells. As seen from our results, [6]-gingerol did not induce any significant cytotoxicity even at 500 micromolar. Lack of induction of apoptosis at these concentrations proved [6]-gingerol to be non-toxic to the normal cells of gastro-intestinal tract. Our results go in hand with a previous report where 900 micromolar [6]-gingerol produced only 50% growth inhibition in rat intestinal epithelial cells [36].Thus [6]-gingerol might be showing differential effects on proliferation of normal cells from different origins, but certainly seems to be safe for use in treating gastro-intestinal disorders.

Protein kinase C (PKC) are group of kinases known to regulate cell growth, differentiation and apoptosis mainly through their ability to phosphorylate and activate their substrates, including the members of MAP kinase family [40], [41]. Phorbol esters such as phorbol-12-myristate-13-acetate (PMA) are known tumor promoters by virtue of their role in activating PKC as substitutes to their physiological activators like diacylglycerol and phosphatidylserine [22], [42]. MAP kinase pathways are involved in regulating proliferation, invasion and metastasis in colon cancer cells [43], [44]. As presented in the results, PMA treatment in SW-480 cells activated the members of MAP kinase family, like ERK1/2, JNK and p38 MAPK, as evident from the transient increase in their phosphorylated forms in Western blotting. Remarkable suppression of this activation upon pre-treatment with [6]-gingerol was very evident in case of ERK1/2 and JNK MAP kinases, but less in the case of p38 MAPK. The results from Western blotting were further confirmed from the cell proliferation assay in presence of the inhibitors of MAP kinases. Reversal of PMA-induced proliferation of SW-480 cells in presence of U0126 and SP600125 provides evidence for the role of ERK1/2 and JNK in the cell proliferation. Also the lack of effect of p38 MAP kinase inhibitor, SB203580, rules out the role of p38 MAP kinase in the process. The combination treatment with [6]-gingerol and inhibitors of ERK1/2 or JNK shows that PMA-induced activation of each of these MAP kinase pathways are down-regulated independent to each other by [6]-gingerol and the additive effects of their down-regulation results in the inhibitory effects seen with [6]- gingerol. Although we have not performed the inhibition of both ERK1/2 and JNK pathways togother in SW-480 cells, we speculate that a double-inhibition would result in inhibition of PMA-induce cell proliferation typically like that with [6]-gingerol alone. Since no additional inhibitory effects of [6]-gingerol were seen in combination with the inhibitors of ERK1/2 or JNK, we believe that no other additional factors up-regulated by PMA in SW-480 cells are affected by [6]-gingerol. Stimulation of MEK-ERK1/2 pathway by growth factors and induction of both ERK1/2 and JNK pathways by oncogenic proteins like Src and Ras, leading to the activation of their downstream targets, like AP-1, have been found crucial in the development of colon cancer [45], [46]. Inhibition of the constituents of MAP kinase pathway has long been recognized as ideal approach to arrest the progression of colon cancer [46].There are reports on other natural compounds like epicatechin gallate, curcumin, silibinin and red ginseng of inducing apoptosis in colon cancer cells via inhibition of MAP kinases [15], [47], [48], [49]. [6]-gingerol was previously shown to inhibit the phosphorylation of ERK1/2, JNK and p38 MAP kinases in mouse skin cancer cell lines, hepatocarcinoma cells and pancreatic cancer cells [28], [34], [36], [50]. But, to the best of our knowledge present study reports for the first time the inhibition of ERK1/2 and JNK MAP kinases as the mechanism of action of [6]-gingerol in reversing the PMA induced proliferation in colon cancer cells.

There are increasing evidence which establish the role of transcription factors like NF-kappB and AP-1 in tumourigenesis, progression, invasion and metastasis of cancer of colon epithelium [46]. In colon cancer, NF-kappaB plays an anti-apoptosis role by many means like, by inhibiting the ROS pathways, by inhibiting JNK cascade and by inducing the expression of anti-apoptotic genes Bcl-2, Bcl-x and cIAPs [51], [52]. NF-kappaB is also known to facilitate angiogenesis, invasion and metastasis in colon cancer tumor cells by up-regulating vascular endothelial growth factor (VEGF), cyclooxygenase 2 (COX-2), interleukine (IL)-6 and matrix metalloproteinases (MMPs) [52], [53]. AP-1 group of transcription factors have a more direct role in tumorogensis of colon cancer. Enhanced activity of AP-1 has been demonstrated in human colon adenocarcinoma and the immune-histochemical analysis of majority of colon adenocarcinoma has revealed high-level expression of AP-1 correlating with the high expression of its downstream targets like EGFR and COX-2 [46]. AP-1 also mediates the anti-apoptotic response to the hypoxic conditions encountered in the solid tumors which contributed to their resistance to chemotherapy and radiotherapy [54]. AP-1 is under the direct regulatory control of MAP kinases, which phosphorylate the Jun and Fos components of AP-1 protein and facilitate their target binding [46], [54].The presence of high concentrations of bile acids also induces the AP-1 expression in colon cells via PKC and ERK1/2 signaling, resulting in tumor promotion [55].Treatment with PMA activates both NF-kappaB and AP-1 transcription factors through Ras/Raf/ERK1/2, JNK and phosphoinositise-3-kinase/Akt signaling pathways [56], [57]. In our study too PMA treatment up-regulated both NF-kappaB and AP-1 in SW-480 cells, as evident from the increase in their transcriptional binding. Pre-treatment with [6]-gingerol drastically reduced the up-regulated AP-1, almost completely abolishing their transcriptional binding at 300 µM concentration. On the contrary [6]-gingerol had only a little effect on the transcriptional binding of NF-kappaB. Inhibition of NF-kappa B with SN50 did not have any significant effects on the PMA-induced cell proliferation of SW-480, suggesting no direct role of the PMA-induced NF-kappaB in enhancing proliferation of SW-480 cells. This goes together with the fact that [6]- ginerol had little effect on PMA-induced NF-kappaB while bringing about cytotoxicity in SW-480 cells. Similar effects were reported previously for inhibitory properties of caffeic acid 3, 4-dihydroxy-phenethyl ester (CADPE) on PMA-stimulated gastric carcinoma cells [57]. CADPE was reported to inhibit the phosphorylation of ERK1/2, with little effects on JNK and p38 MAP kinases, inhibiting the transcriptional binding of AP-1, but with no effect on DNA binding of NF-kappaB. Even though we did not include any inhibitors of AP-1 in studying the inhibition of PMA–induced cell proliferation of SW-480, taken together, our results suggests that AP-1 is down-regulated by [6]-gingerol via the inhibition of phosphorylation of ERK1/2 and JNK MAP kinase in SW80 cells. It is well known that ERK1/2 pathway is involved in activation of the c-Fos component and JNK pathway is involved in activation of both c-Jun and c-Fos components of AP-1 [54]. Also we have seen activation of caspase-3 with inhibition of ERK1/2, JNK MAP kinase and with [6]-gingerol while bringing about inhibition of PMA-induced proliferation of SW-480 cells. Thus, [6]-gingerol must be exerting its effect inhibiting the activation of Ras/Raf/ERK1/2/JNK/AP-1 pathway activated by PMA in SW-480 cells. Compounds and small molecules inhibiting the MAP kinases, AP-1 dimerization or AP-1 transcriptional binding are considered ideal candidates for treatment of colon cancer [46]. Thus [6]-gingerol could be an ideal candidate to be developed as an anti-cancer agent against colon cancer.

Previous works on effect of [6]-gingerol on colon cancer cells describe molecular mechanisms different from the present study. Lee et al., 2008 [13] revealed the inhibition of Cyclin D1 and the activation of NAG-1 as mechanism behind cell cycle arrest and pro- apoptotic effect of [6]-gingerol. Another interesting study from Jeong et al, 2009 [14], demonstrated the inhibition of Leukotriene A4 hydrolase, the terminal enzyme in the Leukoteriene B4 synthesis, as the site of action of [6]-gingerol in inhibiting the anchorage dependent growth of HCT116 colon cancer cells in culture and in xenograft model. Another recent study on inhibitory effect on [6]-gingerol on LoVo cells describes down-regulation of cell cycle regulators Cyclin A, B1 and CDK1 along with the increase in the generation of intracellular reactive oxygen species (ROS) as the mechanism of action [15].The present study identifies a totally different mechanism of action for [6]-gingerol in colon cancer cells.

Down-regulation of AP-1 transcriptional binding by [6]-gingerol directly implies to its therapeutic potential against colon cancer, as AP-1 is known to be the transcription factor responsible for transcription of effectors like COX-2, MMPs and VEGF, which are responsible for invasiveness, metastasis and angiogenesis in colon cancer tumors [46], [54]. [6]-gingerol was previously shown to inhibit COX-2 expression in skin cancer model [28], [29], but till date their effects on COX-2 in colon cancer has not been studied. In the present study, SW-480 being a COX-2 negative cell line [58] might have other down-stream effectors of AP-1, like MMP-9 or VEGF, affected by [6]-gingerol. Down-regulation of MMP-9 and MMP-2 by [6]-gingerol has been reported previously in hepatic cancer and breast cancer cell lines respectively [9], [50].Components of ginger were also shown to inhibit angiogenesis by inhibiting VEGF in ovarian cancer cells [29], [59].Even though the present study has not explored the effect of [6]-gingerol on down-stream targets of AP-1, it calls for attention in exploring its effects on these effectors molecules in colon cancer cells. Extending the results from present study into an *in vivo* study would further provide valuable insights on the bioavailability of [6]-gingerol and its mechanistic role in prevention of colon tumorogenesis.

## Supporting Information

Certificate S1 **Sanction letter of Institutional Animal Ethical Committee.**
(DOCX)
