# Supplementary material for: [6]-Gingerol Induces Caspase-Dependent Apoptosis and Prevents PMA-Induced Proliferation in Colon Cancer Cells by Inhibiting MAPK/AP-1 Signaling
Source: PLoS One. 2014 Aug 26;9(8):e104401. doi: 10.1371/journal.pone.0104401 (PMC4144808; doi:10.1371/journal.pone.0104401)
Supplement: Supplementary file 1 [file pone.0104401.s001.docx]

RAJIV GANDHI CENTRE FOR BIOTECHNOLOGY

An Autonomous National Institute, Government of India
Department of Biotechnology, Ministry of Science & Technology

IAEC/2012/45

27-09-2012

CERTIFICATE

This is to certify that the project titled "**Evaluation of the anticancer effect and toxicity of the active principle of Solanum nigrum (SN2) in in vivo mouse models**" has been approved by the IAEC at the meeting held on 4th June, 2012, at Rajiv Gandhi Centre for Biotechnology.

The Principal Investigator, **Dr.Ruby John Anto** has been given the permission to use the following number of animals for a period of **One year** starting from **20-09-2012** for the above mentioned project as per the protocols submitted and strictly adhering to the CPCSEA rules and regulations.

IAEC File No:	: IAEC/151/RUBY/2012
Species of animal	:Mouse
Strain	: NOD. SCID, Swiss Albino
No: of females	:NOD SCID - 18, Swiss Albino - 54
No: of males	: 0
Total number of animals	:72
Approval date	:20-09-2012
Expiry date	:19-09-2013

Prof. M. Radhakrishna Pillai
Chairman, IAEC
